# Melatonin and Comorbidities in Children with Autism Spectrum Disorder

**DOI:** 10.1007/s40474-018-0147-0

**Published:** 2018-08-09

**Authors:** Katia Gagnon, Roger Godbout

**Affiliations:** 1 Sleep Laboratory & Clinic, Hôpital Rivière-des-Prairies, CIUSSS du Nord-de-l’Île-de-Montréal, 7070 Boul. Perras, Montréal, Québec H1E 1A4 Canada; 2 Department of Psychiatry, Université de Montréal, Montréal, Québec Canada

**Keywords:** Sleep, Anxiety, Pain, Sensory processing dysfunction, Gastrointestinal dysfunction

## Abstract

**Purpose of Review:**

Melatonin is used to treat sleep difficulties associated with autism spectrum disorder (ASD). There are growing evidence that melatonin could have an effect on other symptoms than sleep, such as anxiety, depression, pain, and gastrointestinal dysfunctions. Interestingly, these symptoms frequently are found as comorbid conditions in individuals with ASD. We aimed to highlight the potential effect of melatonin on these symptoms.

**Recent Findings:**

Animal and human studies show that melatonin reduces anxiety. Regarding the effect of melatonin on pain, animal studies are promising, but results remain heterogeneous in humans. Both animal and human studies have found that melatonin can have a positive effect on gastrointestinal dysfunction.

**Summary:**

Melatonin has the potential to act on a wide variety of symptoms associated with ASD. However, other than sleep difficulties, no studies exist on melatonin as a treatment for ASD comorbid conditions. Such investigations should be on the research agenda because melatonin could improve a multitude of ASD comorbidities and, consequently, improve well-being.

## Introduction

Autism spectrum disorder (ASD) is characterized by social and communications impairments as well as by restrictive interests and repetitive behaviors [1] and it occurs in 1 out of 68 children. [2] The current etiological models of autism are multidimensional and based on neurobiological evidence. Several comorbidities are associated with ASD including gastrointestinal disorders, mood disorders, epilepsy, and disruptive behaviors, [3, 4] but poor sleep is one of the most common with rates up to 80%. [5] Poor sleep can have major impact on daytime functioning of individuals with ASD, including increased severity of ASD core symptoms, mental health issues, and challenging behaviors such as tantrums, aggression, and self-injury, [6, 7] as well as parental stress and impaired family well-being. [8] Melatonin is often used to improve sleep in ASD, and there is growing evidence in the general literature that it can also be used to improve symptoms other than sleep. The main objective of this review is to highlight the potential effects of melatonin on sensory processing and several comorbidities associated with ASD.

## Role of Melatonin

Melatonin is a neurohormone secreted by the pineal gland during dark period of the night, [9, 10] and it regulates circadian rhythms including sleep patterns. [11, 12] The pineal gland is, however, not the only structure to secrete melatonin since the gastrointestinal tract, lungs, renal cortex, and the retina have also been found to secrete it, so that it influences the immune system and the reproductive system as well as gastrointestinal motility. [13, 14] Circadian rhythms depend on the endogenous clock situated in the suprachiasmatic nucleus of the anterior hypothalamus but they are also influenced by two classes of environmental factors: photic factors, using the retino-hypothalamic tract, and non-photic factors such as social interaction, exercise and meals schedules.

## Melatonin Release Patterns and Concentration in ASD

Some studies have suggested the presence of abnormal patterns of melatonin release and concentrations in ASD. A pilot study has found for example that while the total nocturnal melatonin urinary concentration was the same in a sample of 10 ASD (mean age 18 ± 2; mean IQ 78) and 10 healthy controls (mean age 35 ± 6; no IQ measure), daytime levels were higher in the ASD group, leading to a decrease in melatonin circadian variation. [15] Nir et al. [16] also reported a higher serum melatonin concentration during daytime in 10 ASD males (age range 16–30 years). They were hospitalized for the last 3 to 24 years and seven of them also suffered from epilepsy. This group was compared to five staff members with the same diet and sleep patterns. In this case, however, the lower amplitude of the melatonin circadian rhythm in the ASD group was due to the fact that they displayed lower nocturnal melatonin concentrations compared to the control group [16] More recent studies on larger cohorts of children, adolescents and post-pubertal individuals have shown a significant lower daytime and nocturnal 6-sulphatoxymelatonin excretion in ASD compared to controls. [17, 18] Interestingly, this was negatively correlated with verbal communication, social play skills, and repetitive use of objects. [17] These studies, however, displayed methodological issues such as the presence of comorbidities, [15, 16, 18] small samples sizes, [15, 16] and variations in melatonin measurement protocols, including the timing of sampling and the nature of samples themselves (blood, urine, saliva) as well as medication use [16, 18]. Other studies have found similar concentration of melatonin in ASD compared to typical developing individuals. [19–21] Again, these studies suffered from methodological weaknesses such as the inclusion of participants with comorbidities, [21] mixing children with adolescents and adults, [20] small sample size [19], and lacking a control group. [19] Variable methods of sampling timing and techniques of dosage varied across studies. We conclude that the melatonin release pattern is not definitively proven to be different in autism using the literature cited above.

### Genetic Studies on Melatonin Levels in ASD

**Fig. 1:**
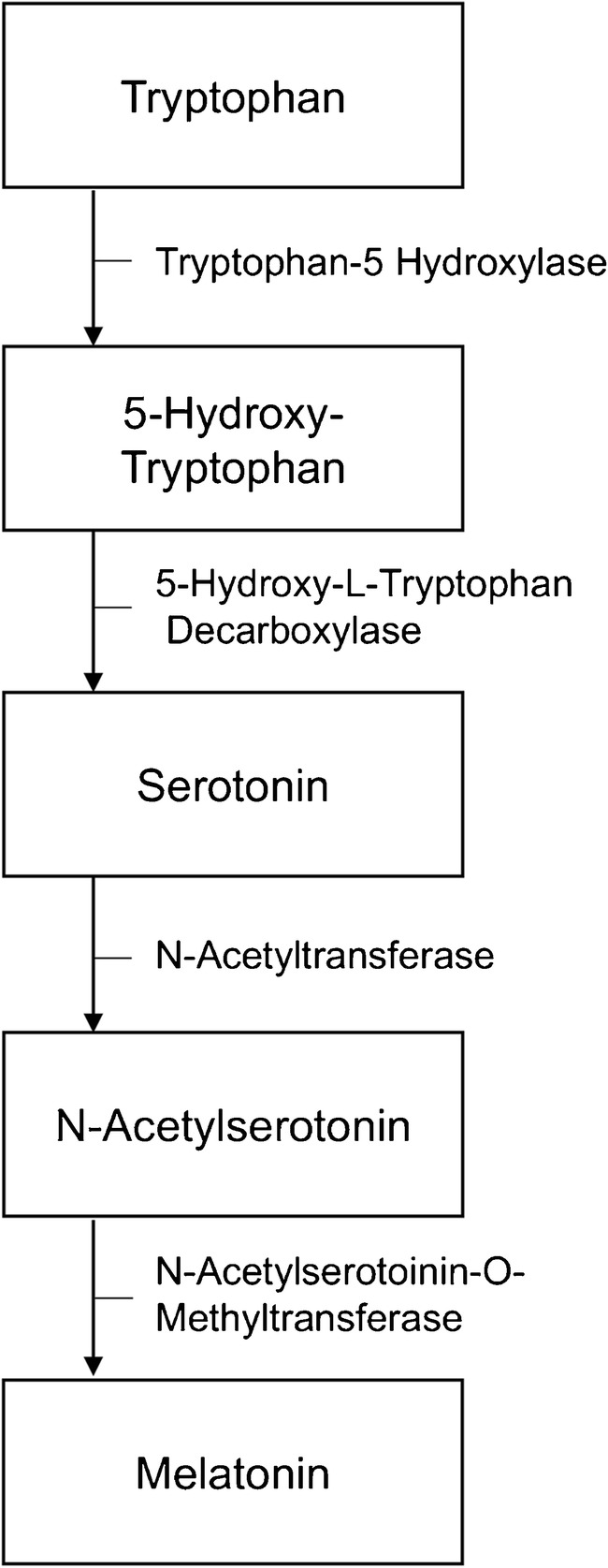
Biosynthesis of melatonin

There is a growing interest regarding the genetics of melatonin in ASD. [22] One hypothesis is that genetic variation impairs the transcription of acetylation and methylation enzymes related to the biosynthesis of melatonin (see Fig. 1). A study by Melke et al. (2008) found lower nocturnal and morning plasma levels of melatonin in a group of 43 adolescents and adult participants with ASD compared to a group of 75 healthy controls. [23] Low melatonin levels were also found in non-affected parents of ASD participants, suggesting a genetic origin. [23] In the same study, mutation screening in 250 independent families of ASD patients compared to 255 control families revealed an enzymatic deficit decreasing the transcript level of acetylserotonin methyltransferase protein (ASMT) which is implicated in the biosynthesis of melatonin. Consistent with these findings, another study from the same group showed that N-acetylserotonin was increased in ASD and inversely correlated with melatonin concentration, suggesting lower transcript levels of ASMT. [24] Another group compared urinary excretion of 6-sulfatomelatonin, the major enzymatic (active) metabolite of melatonin, in 20 ASD children and adolescents according to their serotonin secretion pattern (hyperserotonemic vs normoserotonemic). [25] They found significantly lower 6-sulfatomelatonin secretion in hyperserotonemic compared to normoserotonemic participants, supporting the hypothesis of an impaired serotonin-N-acetylserotonin—melatonin pathway in ASD. We conclude that, using the literature cited above, genetic studies do suggest the existence of low melatonin release in autism.

## Sleep in ASD

People with ASD often experience signs of insomnia such as delayed sleep-onset, fragmented sleep, early morning awakenings, and shortened total sleep. [26] Other types of sleep disturbances are also reported, including parasomnias, sleep-related breathing disorders, sleep-related movement disorders, and circadian rhythm sleep disorders. [26, 27] Parents of children with ASD also report bedtime resistance, which can be linked to factors such as sleep anxiety, sleep-onset association problems, non-compliance, over-activity, or general dysregulation. [27] Objective sleep recordings confirm the subjective reports of poor sleep and can even document signs of poor sleep in non-complaining people with ASD. [28–31] Such studies further document decreased deep slow-waves sleep (stages 3 + 4), less electroencephalographic sleep spindles, and rapid-eye movement sleep abnormalities. [29, 31–33] In conclusion, causes of sleep disturbance in children with ASD are multifactorial, with biological, psychological, and social factors. Melatonin has been used to improve sleep in children with ASD, but its mechanism of action remains unclear. The rest this review will discuss the growing evidence that melatonin can act on ASD symptoms and comorbidities such as anxiety, pain/sensory processing, and gastrointestinal disorders, which may be responsible for sleep problems in ASD.

## Anxiety in ASD

ASD is associated with a high risk for anxiety disorders, which can exacerbate ASD core symptoms and challenging behaviors. [34] According to a recent meta-analysis, 39.6% of young individuals with ASD had clinically elevated anxiety with at least one anxiety disorder. [35] The most common anxiety disorders in ASD are specific phobia (29.8%), obsessive compulsive disorder (17.4%), and social anxiety and agoraphobia (16.6%). [35].

The clinical presentation of anxiety in ASD may be different from that in the general population. [34] Anxiety can manifest itself with heterogeneous features in people with ASD, including fears of novelty and worries concerning special interests and unusual phobias. [36] Repetitive behaviors such as stereotyped (i.e., flapping, rocking) or more complex behaviors (i.e., following the same routine) can be comforting for ASD individual but can become a source of anxiety when interrupted. [37] It has thus been suggested that anxiety and ASD share the same etiology, while others posit that anxiety could be a consequence of the interactions between ASD individuals and their environment. [34] Impairments in social interactions cause anxiety because ASD individuals may not be able to understand or predict the actions of their neurotypical counterparts, and consequently meet social expectations. [34] Atypical sensory processing in ASD (i.e., hypersensitivity to sensory stimuli such as sounds) also leads to anxiety in ASD individuals and for example prevent them from going to movie theaters, because of very loud sound, while they really enjoy watching movies at home.

### Anxiety Contributes to Sleep Problems in ASD

The link between sleep problems and anxiety is clearly demonstrated in typically developing children. [38] The prevalence of insomnia in individuals with anxiety disorders is 70 to 90%. [39] Sleep problems are also relatively common in ASD youth with comorbid anxiety. [40] Paavonen et al. [41] found a high prevalence of sleep-related fears and negative attitudes towards sleep that could be due to anxiety levels as previously proposed by Richdale et al. [42]. Sleep studies in adults with ASD also showed that anxiety is associated with poor sleep in ASD. [29, 43] Others have identified a positive correlation between sleep problems and anxiety among children with intellectual disability and/or ASD. [44] Even though the relationship between ASD and anxiety alone was not investigated, the authors found that the presence of sleep problems and anxiety was a strong predictor of challenging behaviors during the day. A study on sleep and psychopathology in 17 ASD children and 15 typically developing children showed that poor sleep was related to anxiety in both groups but the type of anxiety differed: in ASD children, poor sleep was associated with somatic-panic, whereas school, separation, and general anxiety emerged in neurotypical children. [45] A recent study with a large cohort of 1347 ASD children and adolescent found that anxiety was associated with all types of sleep problems, including bedtime resistance, sleep-onset delay, sleep duration, sleep anxiety, and night waking. [46•] This study also reported a positive association between anxiety and sensory sensitivity. [46•].

### Melatonin and Anxiety

There is a growing interest regarding melatonin as a new treatment approach for anxiety. Recently, animal studies have focused on the effect of melatonin on chronic stress and found that melatonin displayed long-term anxiolytic effects and suppressed anxiety-like behaviors. [47–49] Furthermore, melatonin was associated with improved working memory and reduced systemic responses to stress. Another study in rats investigated anxiety following 72 h of sleep deprivation and found that melatonin prevented the anxiety-like behaviors induced by sleep deprivation. [50] Using a transgenic mouse model of Alzheimer’s disease, Nie et al. (2017) showed that melatonin reduced proteins associated with anxiety (glutathione S-transferase P 1) and depression (Complexin–1). [51] Liu et al. (2017) focused on the role of melatonin in the pathophysiology of mood disorder. Using a transgenic mouse model, they found that the deletion of MT1 and/or MT2 melatonin receptors was associated with deficits in hedonic and social interaction behaviors and increased anxiety-like behaviors, similar to some of the core symptoms of ASD. [52]

Clinical trials in humans have shown that melatonin reduces pre-operative anxiety. [53•] Recent studies investigated the efficacy of melatonin in pre-operative anxiety and post-operative pain compared to standard anxiolytic/sedative medication. Studies with children found that melatonin (0.5 mg/kg, p.o.) was as effective as midazolam in reducing pre-operative anxiety. [54, 55] With higher doses (0.75 mg/kg), melatonin was found to be more effective than midazolam for pre-operative anxiety without causing sedation and cognitive dysfunctions as midazolam did. [55] Patel et al. (2015) found similar results with adults, whereas Caumo et al. (2009) showed that melatonin was as effective as clonidine in reducing post-operative anxiety. [56, 57]

Based on these results and because anxiety is associated to all type of sleep problems in children with ASD, oral melatonin could be considered as an effective means to reduce anxiety and promote sleep. However, there are no studies on the use of oral melatonin to specifically improve anxiety in children with ASD, and such investigations should rank high on the research agenda.

## Sensory Processing Dysfunction

Sensory processing problems are frequent in ASD, with an estimated rate of 90% and it is now a diagnostic feature of ASD. [58] According to the *Diagnostic and Statistical Manual of Mental Disorders* (5th edition), the symptomatology is defined as a hyporesponsivity or a hypersensitivity to sensory stimulation, or by an unusual interest in sensory aspect of the environment. [1] Hence, there are three main sensory patterns in patients with ASD: hypo-responsiveness, hyper-responsiveness, and sensory seeking. [59] Between 56 and 69% of children experience sensorial hypersensitivity, also called hyper-responsiveness. [60, 61] As discussed earlier in this review, sensory hyper-responsiveness is an ASD symptom that can contribute to anxiety and consequently to sleep disorders. In fact, sensory hyper-responsiveness has been associated with hyperarousal, which has an impact on sleep. [46•] Moreover, children with ASD may experience pain or discomfort related to sensory processing dysfunction, for example, discomfort related to temperature, clothing texture, or noise. [62] Children might also experience chronic pain (e.g., associated with gastrointestinal dysfunction) or mild illnesses (e.g., sore throat or rhinitis) but the difficulty ASD children have reporting their sensory discomfort to their caregivers is an issue. It is also important to note that the relationship between sleep and sensory processing is bidirectional: sensory discomfort increases the risk of sleep problems and sleep problems influence sensory processing. [63, 64].

### Sensory Problems and Sleep

Studies suggest a link between sensory hyper-responsiveness and sleep difficulties. A study in 56 typically developing schoolchildren found that higher tactile, auditory, and movement sensitivity was a significant predictors of poor sleep. [65] Children with ASD have been found to be more sensitive to their environment (complain that the room or bed are uncomfortable; awakened or frightened by noises) compared to children with intellectual disability, developmental disabilities, and controls. [65] A study including 27 children with ASD and 28 neurotypical controls showed that sensory-avoiding behaviors and physiological markers of stress were highly correlated with sleep problems in children with ASD. [66] A large cohort study with 1347 children and adolescents found that hyper-responsiveness in ASD participants aged between 2 and 5 years was associated with sleep-onset delay, short sleep duration, and night awakenings. Among ASD participants aged between 6 and 12 years, sensory hyper-responsiveness was associated with bedtime resistance, sleep anxiety, sleep-onset delay, and sleep duration. [46•]

### Melatonin and Sensory Modulation

To our knowledge, no studies have yet investigated the effect of melatonin on sensory processing dysfunction. Since melatonin can reduce pain perception, [67] it could possibly improve sensory hyper-responsiveness. Studies in rodents have investigated the relationship between melatonin and pain in conditions such as acute pain, inflammatory pain, and neuropathic pain. [68] Melatonin was shown to have a significant anti-nociceptive effect in all experimental models of pain. [68] Moreover, melatonin has anti-oxidative and anti-inflammatory properties, reducing pain related to tissue damage. [68]

The exact mechanism underlying the analgesic effect of melatonin is still unknown but it has been proposed that exogenous melatonin could exert its anti-nociceptive effect either directly via the activation of central MT1 and MT2 receptors located in the brain and spinal cord or indirectly via opioid, GABA, or NMDA receptors or potassium and calcium channels. [68] The improvement of sleep, the sedative effects, and the anxiolytic properties of melatonin could also play an indirect, modulatory role (see above) on its effects on pain. Recent animal studies on neuropathic pain, fibromyalgia, orofacial pain, and allodynia (painful sensation caused by an innocuous stimulus) have also documented the analgesic effects of melatonin. [69–74] Such studies indicate that melatonin could reduce inflammatory and oxidative stress by modulating the nitroxidergic system. Lin et al. (2017) have identified MT2 melatonin receptors in the dorsal root ganglia as being involved in the melatonin analgesic effect on neuropathic pain. [72] Interestingly, another group has recently reported that a single intraperitoneal injection of melatonin (50 mg/kg) reduced mechanical and thermal hyperalgesia induced by an orofacial pain model for up to 7 days. [73]

Studies on the effectiveness of melatonin on pain in human are controversial. Most randomized, double-blind, placebo-controlled studies have administered various doses of melatonin before a medical procedure (i.e., intubation, hysterectomy, prostatectomy, ophthalmologic procedure, laparoscopic cholecystectomy) and patients were anesthetized. Post-operative pain was assessed with questionnaires and use of standard analgesics. Results of such studies are heterogeneous, some showing a significant reduction of post-operative pain while others did not. [53•, 68] For example, a study aimed to assess the analgesic efficacy of melatonin (3 and 6 mg) upon cesarean section under spinal anesthesia found no significant differences between groups for the duration of anesthesia and for analgesia. [75] A bariatric surgery study assessed the effects of melatonin premedication (5 mg) on post-operative recovery and found an improvement of sleep and reduced pain. [76] A study on the effect of melatonin premedication (6 mg) following the extraction of a third molar (“wisdom tooth”) found a significant effect on post-operative pain and anxiety in female but not in male subjects. [77] Chronic melatonin (4 mg at bedtime) was used as a prophylactic therapy in 41 patients aged 18–75 years suffering from tension-type headaches or migraine. The frequency of headaches was decreased at the 6-month follow-up but data are not shown for the 2-month follow-up measures. [78]

There are several explanations for these heterogeneous melatonin results. First, pain sensation for the same stimulus can vary widely from one individual to another. Consequently, pain threshold or tolerance should be determined for each participant before the intervention but this is not always done. The study of Stefani et al. (2013) did take such a measure in 61 healthy volunteers (age range 19–47 years) and found that acute, exogenous melatonin (0.05, 0.15, or 0.25 mg/kg sublingual) could increase pressure and heat pain threshold/tolerance. [79] A second issue is that doses of melatonin vary widely from study to study and Stefani et al. (2013) showed a significant correlation between melatonin plasma concentration and the analgesic effect. [79] Thirdly, sample sizes of many studies are small, increasing the risk of type II error even more in a context where inter-individual variability for pain perception is high. Finally, there are sex differences between males and females in the effectiveness of melatonin in reducing pain, as shown by Seet et al. [77]

Animal studies show promising results regarding the effectiveness of melatonin on pain sensation. Indeed, melatonin modulates both ascending pain pathways, namely lemniscal and spinothalamic pathways, involved in acute and chronic pain. [80] Because the lemniscal ascending pathway is also involved in sensorial perception, such as touch and proprioception, melatonin could be a good candidate to modulate sensory processing in children with ASD, but the research still needs to be done.

## Gastrointestinal Dysfunction in ASD

According to a recent review, almost 50% of individuals with ASD complain of gastrointestinal discomfort, but due to a lack of precision and validity in measurement of gastrointestinal symptoms in ASD across studies, the prevalence ranges from 4.2 to 96.8%. [81] There is also a wide range of gastrointestinal problems reported among ASD individuals, including feeding abnormalities, constipation, diarrhea, alternating diarrhea and constipation, abdominal pain or discomfort, gastroesophageal reflux or heartburn, bloating, gas or flatulence, incontinence, pain or difficulty having a bowel movement, and colic. [81] The gastrointestinal symptoms in ASD with the highest prevalence are constipation (22%), chronic constipation (19.7%), chronic diarrhea (16.2%), abdominal pain discomfort (14%), and intermittent diarrhea (13%). [81]

Several hypotheses have been suggested to understand the etiology of gastrointestinal problems in individual with ASD. According to a recent review, gastrointestinal dysfunction in ASD could be the consequence of an underlying inflammatory process. [82] Another hypothesis is that a significant proportion of children with ASD and chronic gastrointestinal problems are suffering from irritable bowel syndrome which is characterized by a group of symptoms, including diarrhea and/or constipation, and abdominal pain or discomfort which are relieved with the passage of stools. [82] It has been suggested that gastrointestinal symptoms were related to cortisol response to stress in ASD. [83] More recently, studies have focused on the gut microbiome as the cause of gastrointestinal disorders in ASD because it contributes to the pathophysiology of many gastrointestinal dysfunctions, including inflammatory bowel disease, functional gastrointestinal disease, and food allergy. [82, 84] There is indeed growing evidence that individuals with ASD have an abnormal gut microbiota. [85••] A study investigating fecal yeast and microbiota in ASD found an increase of *Candida albicans* among ASD participants. [86] They identified that the microbiota composition in ASD was less rich compared to controls. Moreover, the presence of inflammation was correlated with ASD symptom severity. [86] Luna et al. (2017) compared the microbiome profile of ASD and neurotypical children with and without gastrointestinal disorder using rectal biopsies and blood samples. [87] They found distinct microbiome and inflammatory cytokines in the rectal mucosa of ASD children with gastrointestinal disorder. Moreover, the tryptophan level was higher in ASD children with abdominal pain. This finding suggests that once again the serotonin pathway could be involved. (see Fig. 1) Rose et al. (2017) verified whether mitochondrial dysfunction could contribute to gastrointestinal dysfunction in children with ASD. [88] They compared rectal and caecum biopsies between 10 ASD children with gastrointestinal complaints to 10 children with Crohn’s disease and 10 children with non-specific gastrointestinal complaints. The results showed abnormalities in the gut mucosa mitochondrial activity in children with ASD that differed from the other two groups.

Studies have evaluated treatment efficacy of improving the gut microbiota in children with ASD. [85••, 89] Shaaban et al. (2017) ran a prospective open-label study exploring the efficacy and tolerability of 3 months supplementation of probiotics in 30 ASD children aged between 5 and 9 years. [89] They found a significant improvement in the severity of ASD core symptoms as well as gastrointestinal symptoms compared to baseline data. In a recent open-label clinical trial, Kang et al. (2017) used microbiota transfer therapy with 18 ASD children with gastrointestinal dysfunctions. [85••] The protocol included 2 weeks of antibiotic, a bowel cleanse, and a fecal microbiota transplant with maintenance dose over 8 weeks. A significant improvement of gastrointestinal symptoms, ASD core symptoms, and microbiota diversity persisted after the 8-week treatment.

### The Impact of Gastrointestinal Dysfunction on Sleep in ASD

Only a few studies have focused on the relationship between gastrointestinal dysfunction and sleep in children with ASD. A study by Yang et al. (2018), with 169 autistic children and 172 healthy controls aged between 3 and 12 years, found that the proportion of comorbid gastrointestinal and sleep disorders was significantly higher in children with ASD. [90] Conversely, a study with a smaller sample (35 children with ASD and 31 healthy controls) did not find a significant association between gastrointestinal problems and autism symptom severity. [91] Another study including 610 children with ASD aged between 2 and 18 years showed that gastrointestinal disorder was a risk factor for poor sleep. After controlling for several variables, such as age, gender, behaviors, bed-wetting, and medication, the authors found that the presence of gastrointestinal disorder in ASD children increased the risk of having multiple sleep disorder symptoms. [92] Studies suggest that treatment of gastrointestinal disorder could reduce the severity of sleep problems and improve quality of life in ASD individuals and their families. However, further investigations are needed to determine whether treatment of gastrointestinal symptoms in children with ASD reduces the severity of sleep problems.

### Melatonin and Gastrointestinal Dysfunction

The neuroendocrine cells of the pineal gland are not the only cells to produce melatonin: the enterochromaffin cells, which are enteroendocrine cells located in the digestive mucosa, also do, using the same receptors as in the brain. [9] Circadian variation in gastrointestinal melatonin is regulated by food intake and composition. [93] Compared to the blood and the pineal gland, melatonin has a higher concentration in the gastrointestinal tract, [94] which suggests that melatonin could play a major role in gastrointestinal functioning. Indeed, melatonin appears to influence the gastrointestinal system in several ways: it modulates motility regulation by exerting both excitatory and inhibitory effects on gut muscles. In fact, low-dose melatonin increases intestinal motility, while a high dose of melatonin decreases intestinal transit in rats. [95] Melatonin could also modulate the inflammatory response. Studies showed that melatonin reduced the severity of intestinal inflammation in animal models of colitis. [96, 97] Melatonin may also have analgesic effects (see above) so it could modulate visceral sensations and reduce abdominal pain. A study of 40 neurotypical adult participants with sleep disturbances and irritable bowel syndrome found that 3 mg of melatonin at bedtime for 2 weeks decreased abdominal pain and increased pain threshold. [98] Because patients with irritable bowel syndrome often complains of psychological symptoms such as stress, anxiety, and depression, [99] authors suggested a brain-gut interaction that could influence gastrointestinal symptoms. Since evidence shows that melatonin can have anxiolytic effects (see above), it could also improve the relationship between stress/anxiety and gut motility. However, clinical studies on mood, including anxiety and depression, and irritable bowel syndrome show mixed results. [99] In addition of being frequently reported in patients with irritable bowel syndrome, poor sleep is also known as a risk factor for gastrointestinal symptoms. [100]

Sleep, circadian rhythms, and melatonin also play a role in the regulation of gastrointestinal tract inflammation. [101] A pilot study including 38 women used blood samples and complete polysomnography to demonstrate a significant reduction of melatonin/tryptophan levels in patients with diarrhea and irritable bowel syndrome compared to those with constipation and irritable bowel syndrome, and healthy controls. [102] The authors concluded that low nighttime melatonin levels could be involved in the etiology of sleep disturbance in individuals with diarrhea and irritable bowel syndrome. An animal model of colitis has demonstrated that sleep deprivation increases inflammation and leads to weight loss. [103] Using a mouse model of colitis and sleep deprivation, Park et al. (2015) found that melatonin significantly reduced pro-inflammatory cytokines, reduced weight loss, and increased survival. [103]

Esteban-Zubero et al. (2017) recently proposed that melatonin treatment could improve quality of life and decrease pain related to irritable bowel syndrome. [104] According to a randomized, placebo-control study with 80 postmenopausal women with irritable bowel syndrome, 6 months of melatonin (5 mg at bedtime and 3 mg in the morning) showed a significant improvement of constipation symptoms relative to placebo in 60% of participants, whereas only 45% of participants with diarrhea improved, which did not significantly differ from the placebo treatment. [105] Interestingly, a placebo-controlled, randomized study using probiotics as a treatment for irritable bowel syndrome demonstrated that symptom improvement was correlated with an increase in morning melatonin. This result suggests that probiotics could influence melatonin production. [106]

Gastrointestinal dysfunction has a negative impact on sleep in children with ASD. [90, 92] Melatonin treatment could improve gastrointestinal symptoms in people with ASD because it may improve not only improve gastrointestinal motility, but also ameliorate sleep, reduce pain, and anxiety. Unfortunately, no studies have investigated the effect of melatonin treatment on gastrointestinal dysfunctions in ASD.

## Conclusion

Many studies have investigated the role of melatonin on sleep in ASD. There is now a growing literature suggesting that melatonin could also have a positive effect on other mental and physical health problems often associated with ASD, such as anxiety, pain/sensory processing, and gastrointestinal dysfunction. The purpose of this review was to highlight the potential effect of melatonin on these health issues. Briefly, animal and human studies have shown that melatonin improves anxiety and gastrointestinal dysfunction, whereas studies in animals have demonstrated positive results on sensory processing but results in humans are heterogeneous. Anxiety, sensory processing dysfunction/pain, and gastrointestinal problems are known to have a negative effect on sleep. It is thus reasonable to think that melatonin could at least partially draw some of its positive effects on sleep in ASD by acting on these alternative routes.
